# G-Protein Signaling Modulator 2 as a Potential Biomarker in Colorectal Cancer: Integrative Analysis Using Genetic Profiling and Pan-Cancer Studies

**DOI:** 10.3390/genes15040474

**Published:** 2024-04-09

**Authors:** Doaa Jawad Kadhim, Hanieh Azari, Saeideh Khorshid Sokhangouy, Seyed Mahdi Hassanian, Hawraa Ibrahim Alshekarchi, Ladan Goshayeshi, Lena Goshayeshi, Mohammad Reza Abbaszadegan, Fatemeh Khojasteh-Leylakoohi, Majid Khazaei, Ibrahim Saeed Gataa, Godefridus J. Peters, Gordon A. Ferns, Jyotsna Batra, Alfred King-Yin Lam, Elisa Giovannetti, Amir Avan

**Affiliations:** 1 Metabolic Syndrome Research Center, Mashhad University of Medical Sciences, Mashhad 91779-48564, Iranazari.hanie@gmail.com (H.A.); hasanianmehrm@mums.ac.ir (S.M.H.); fatemekhjst@gmail.com (F.K.-L.); khazaeim@mums.ac.ir (M.K.); 2 Medical Genetics Research Center, Mashhad University of Medical Sciences, Mashhad 91886-17871, Iran; saeedehkhorshid899@gmail.com (S.K.S.); abbaszadeganmr@mums.ac.ir (M.R.A.); 3 Al-Zahraa Center for Medical and Pharmaceutical Research Sciences (ZCMRS), Al-Zahraa University for Women, Kerbala 56001, Iraq; 4 Department of Gastroenterology and Hepatology, Faculty of Medicine, Mashhad University of Medical Sciences, Mashhad 91779-48564, Iran; goshayeshil@mums.ac.ir; 5 Surgical Oncology Research Center, Mashhad University of Medical Sciences, Mashhad 91779-48954, Iran; 6 College of Medicine, University of Warith Al-Anbiyaa, Karbala 56001, Iraq; 7 Department of Biochemistry, Medical University of Gdansk, 80-211 Gdansk, Poland; gj.peters@amsterdamumc.nl; 8 Cancer Center Amsterdam, Cancer Biology and Immunology, Amsterdam UMC, Vrije Universiteit, Department of Medical Oncology, 1081 HV Amsterdam, The Netherlands; 9 Department of Medical Education, Brighton & Sussex Medical School, Falmer, Brighton BN1 9PH, UK; g.ferns@bsms.ac.uk; 10 Faculty of Health, School of Biomedical Sciences, Queensland University of Technology (QUT), Brisbane, QLD 4059, Australia; jyotsna.batra@qut.edu.au; 11 Pathology, School of Medicine and Dentistry, Gold Coast Campus, Griffith University, Gold Coast, QLD 4222, Australia; a.lam@griffith.edu.au; 12 Cancer Pharmacology Laboratory, AIRC Start Up Unit, Fondazione Pisana per La Scienza, 56017 Pisa, Italy

**Keywords:** GPSM2, colorectal cancer, TCGA, pan-cancer

## Abstract

Colorectal cancer (CRC) imposes a significant healthcare burden globally, prompting the quest for innovative biomarkers to enhance diagnostic and therapeutic strategies. This study investigates the *G-protein signaling modulator (*GPSM) family across several cancers and presents a comprehensive pan-cancer analysis of the *GPSM2* gene across several gastrointestinal (GI) cancers. Leveraging bioinformatics methodologies, we investigated *GPSM2* expression patterns, protein interactions, functional enrichments, prognostic implications, genetic alterations, and immune infiltration associations. Furthermore, the expression of the *GPSM2* gene was analyzed using real-time analysis. Our findings reveal a consistent upregulation of *GPSM2* expression in all GI cancer datasets analyzed, suggesting its potential as a universal biomarker in GI cancers. Functional enrichment analysis underscores the involvement of *GPSM2* in vital pathways, indicating its role in tumor progression. The prognostic assessment indicates that elevated *GPSM2* expression correlates with adverse overall and disease-free survival outcomes across multiple GI cancer types. Genetic alteration analysis highlights the prevalence of mutations, particularly missense mutations, in *GPSM2*. Furthermore, significant correlations between *GPSM2* expression and immune cell infiltration are observed, suggesting its involvement in tumor immune evasion mechanisms. Collectively, our study underscores the multifaceted role of *GPSM2* in GI cancers, particularly in CRC, emphasizing its potential as a promising biomarker for prognosis and therapeutic targeting. Further functional investigations are warranted to elucidate its clinical utility and therapeutic implications in CRC management.

## 1. Introduction

Colorectal cancer (CRC) represents a significant and urgent public health issue worldwide, with its incidence on the rise over the years [1]. It is recognized as the third most common cancer worldwide, with projections indicating a surge to 3.2 million new cases annually by 2040 [2]. In 2020 alone, approximately 0.94 million deaths were attributed to CRC [1,2]. The origins of sporadic CRC can be broadly categorized into two types: hereditary or sporadic. Sporadic CRC is the predominant form, accounting for over 80% of cases [3]. This subset of CRC is characterized by chromosomal instability, microsatellite instability (MSI), and CpG island methylation [4]. The development of sporadic CRC is influenced by a combination of somatic genetic and epigenetic events. [5].

Each CRC patient has a unique genetic profile, contributing to the diverse heterogeneity among CRC patients [6]. The advent of next-generation sequencing (NGS) has confirmed previously identified genetic alterations and classified new alterations in sporadic CRCs. The development of new prognostic biomarkers for sporadic CRC is crucial for improving patient outcomes [7]. The Cancer Genome Atlas (TCGA) has played a significant role in identifying genetic alterations in sporadic CRC, which can subsequently lead to the application of new treatment approaches [8]. The ongoing efforts to develop new prognostic biomarkers, such as those using NGS and TCGA, offer hope for improved early detection of recurrence and treatment outcomes for individuals affected by sporadic CRC. These advancements in biomarker research hold promise for personalized and more effective treatment strategies in the management of CRC.

Cancer is frequently linked to disruptions in cellular signaling pathways. For example, the overactivation of pathways such as WNT-β-catenin, TGFβ, PI3K, or RAS can significantly influence various biological processes, including essential functions like cell survival, proliferation, and migration [9,10]. One key component of these pathways is G proteins, formally known as guanine nucleotide-binding proteins, which play a crucial role in transmitting signals from outside the cell within the cellular environment [11]. These internal molecular switches are initiated by G protein-coupled receptors located at the cell membrane, leading to alterations in cellular functionality [11,12]. The activity of G proteins is further managed by another class of proteins, termed G protein-signaling modulators (GPSMs), which interact with subunits of G proteins. GPSMs act as receptor-independent activators of G protein signaling, with notable members including *GPSM1*, *GPSM2*, *GPSM3*, and *GPSM4* (*PCP2*) [13,14].

*G protein-signaling modulator 2* (*GPSM2*) has been identified as a crucial factor in the progression of cancer [15]. The involvement of *GPSM2* in cancer can be understood through several aspects: Initially, *GPSM2* is frequently overexpressed in most tumors versus healthy controls. Its transcript and protein levels typically increase significantly in most tumors, indicating a cancer-promoting role for *GPSM2* [15,16]. Secondly, increased expression of *GPSM2* has been linked to a poorer prognosis in cancer patients. For instance, in liver hepatocellular carcinoma (LIHC), increased *GPSM2* expression was correlated with decreased overall survival (OS) and disease-free survival (DFS) [17]. Additionally, *GPSM2* contributes to maintaining cell polarity and spindle orientation during mitosis, which could potentially influence tumor growth and progression [18]. Furthermore, *GPSM2* has the capacity to modulate immune cell infiltration in the tumor microenvironment and promote tumor cell migration. It plays a role in regulating immune cell levels and facilitating cancer cell movement [15,19]. Lastly, the expression and function of *GPSM2* appear to vary depending on the type of tumor. For example, in non-small cell lung cancer tissues, *GPSM2* was found to be downregulated, and the silencing of *GPSM2* enhances the metastatic potential of cancer cells in both in vitro and in vivo environments. [20]. Moreover, evidence suggests that overexpression of *GPSM2* in breast tumors has an adverse prognostic value in terms of nuclear expression. Furthermore, *GPSM2* appears to negatively affect the efficacy of paclitaxel, a commonly used and well-tolerated chemotherapeutic agent for breast cancer [21].

Despite significant advancements in identifying prognostic markers for CRC, the disease remains a leading cause of mortality and morbidity worldwide, and its molecular underpinnings remain elusive. Recent investigations have highlighted the importance of the GPSM family, particularly *GPSM2*, in various cancer types. However, to the best of our knowledge, there has been limited exploration of the GPSM family’s contribution to CRC. Consequently, this study aims to delve deeper into the role of the GPSM family in CRC by employing bioinformatics approaches and leveraging next-generation sequencing technologies. Through these methods, we seek to identify *GPSM2* as a potential diagnostic and prognostic candidate in CRC.

## 2. Materials and Methods

### 2.1. Data Collection and Patient Samples

Here, we employed two distinct case–control groups. The first group consisted of participants from the COAD-TCGA dataset (http://cancergenome.nih.gov/) (accessed on 23 January 2024). The second group was established for validation purposes, focusing on chemotherapy-naive patients who received treatment at the Omid Hospital of Mashhad University of Medical Sciences. The study was approved by the local Ethics Committee of Mashhad University of Medical Sciences.

### 2.2. FireBrowse

The FireBrowse database (http://firebrowse.org) (accessed on 23 January 2024) provides a user-friendly interface to the Broad Institute’s GDAC Firehose analysis pipeline, offering access to The Cancer Genome Atlas (TCGA) data [22]. This database facilitates the exploration of comprehensive cancer genomics data, including clinical annotations, DNA copy number variations, microRNA (miR) data, and RNA sequencing data, among others. To investigate the expression of four GPSM family members (*GPSM1*, *GPSM2*, *GPSM3*, and *PCP2*) across different cancer datasets, including colon adenocarcinoma (COAD), we utilized this database.

### 2.3. UALCAN

The The University of ALabama at Birmingham CANcer data analysis Portal (UALCAN) (http://ualcan.path.uab.edu) (accessed on 23 January 2024) enables the computational analysis of transcriptome sequencing data obtained from the TCGA initiative [23]. By utilizing UALCAN, we conducted analyses on the relative expression levels of *GPSMs* across normal and CRC specimens, including comparisons between different stages and subtypes of tumors.

### 2.4. RNA Sequencing Data Processing and Differential Expression Analysis

R packages such as DESeq2 and edgeR were employed to identify differentially expressed mRNAs (DEmRNAs) between COAD tissues. To ensure robust and convincing results in a large sample size, we have set a false discovery rate (FDR) threshold of less than 0.01 and a fold change threshold of greater than 1.5 for identifying differential RNA expression.

### 2.5. Pan-Cancer Analysis of GPSM2 in Gastrointestinal Cancers

The Pan Cancer study was conducted to investigate the role of *GPSM2* in gastrointestinal cancers.

We conducted an analysis of the differential expression of *GPSM2* in various gastrointestinal tumors, including colon adenocarcinoma (COAD), esophageal carcinoma (ESCA), pancreatic adenocarcinoma (PAAD), rectal adenocarcinoma (READ), gastric adenocarcinoma (STAD), and relevant normal tissues from the TCGA database, utilizing TIMER2 data. Subsequently, we employed Gene Expression Profiling Interactive Analysis 2 (GEPIA2) (http://gepia2.cancer.pku.cn/) (accessed on 23 January 2024) to generate box plots from the GTEx database [24,25]. Our methodology involved setting a *p*-value cutoff of 0.05, a log2 fold change (FC) cutoff of 1, and ensuring the matching of TCGA normal and GTEx data. GEPIA2 was then used to assess the protein level of *GPSM2* in gastrointestinal cancers (GI). Additionally, we analyzed *GPSM2* expression at different pathological stages of GI cancers using GEPIA2. To obtain expression data and create violin plots, we utilized the log2 [transcripts per million (TPM)+1] on the log scale.

In the next step, the expression levels of GPSM2 in normal gastrointestinal tissues were obtained from the Human Protein Atlas (HPA) database. Subsequently, the STRING database (https://string-db.org/) (accessed on 23 January 2024) was employed to identify proteins that interact with GPSM2. In this study, “GPSM2” was entered into the STRING database, with “homo sapiens” selected as the species, and “medium confidence (0.4)” was chosen as the confidence level. The identified genes were then used to conduct functional enrichment analysis using Gene Ontology (GO) in Enrichr (https://maayanlab.cloud/Enrichr/) (accessed on 23 January 2024) and Kyoto Encyclopedia of Genes and Genomes (KEGG) analyses. The “clusterProfiler” package in R version 4.2.2 was utilized for the enrichment analysis.

We utilized GEPIA2 to conduct survival analysis on TCGA datasets. The overall survival (OS) and disease-free survival (DFS) for GPSM2 across all gastrointestinal (GI) cancers were determined using the “Survival Analysis” module in GEPIA2. Subsequently, OS and DFS were calculated, and Kaplan–Meier plots were generated for each GI cancer individually. The log-rank *p*-value, 95% confidence intervals, and hazard ratio (HR) were computed, with significance levels set at *p* < 0.05. Additionally, the Kaplan–Meier plotter (http://kmplot.com/analysis/) (accessed on 23 January 2024) was employed for analyzing OS, RFS, PPS, and PF across various Gene Expression Omnibus (GEO) datasets. Kaplan–Meier survival plots for colon, gastric, and pancreatic cancers were produced by inputting “GPSM2” into the “mRNA gene chip” module.

We also utilized GEPIA2 for survival analysis on TCGA datasets. To examine the genetic alterations of *GPSM2* in gastrointestinal (GI) cancers, we accessed the cBioPortal database (https://www.cbioportal.org/) (accessed on 23 January 2024). Initially, we selected “colorectal adenocarcinoma” and “Esophagus/Stomach” in the “Quick Select” section for our query. Subsequently, we entered “*GPSM2*” in the gene query box. We downloaded the mutation type, alteration frequency, and copy number alteration (CNA) data across various GI datasets from the “Cancer Types Summary” module. Additionally, we obtained the survival analysis for all GI cancer samples, both with and without GPSM2 genetic alterations, from the “Comparison/Survival” module.

To analyze the correlation between *GPSM2* expression and immune infiltration in gastrointestinal (GI) cancers, we employed the Tumor Immune Estimation Resource (TIMER) web-based tool, accessible at https://cistrome.shinyapps.io/timer/ (accessed on 23 January 2024). We entered “*GPSM2*” into the gene symbol query box. For the cancer types, we selected COAD, ESCA, PAAD, READ, and STAD to evaluate the presence of immune infiltrating cells, including B cells, CD4+ T cells, CD8+ T cells, macrophages, neutrophils, and dendritic cells. Subsequently, in TIMER2, we utilized the immune association module to investigate the relationship between *GPSM2* expression and CD8+ T cell infiltration. We also chose the “Purity Adjustment” option for the Spearman correlation analysis. The results for the various GI cancer types are presented in a heatmap.

### 2.6. DNA-Seq and Whole Exome Sequencing

Somatic mutations of the *GPSM2* gene were identified from the TCGA database using the Mutation Annotation Format (MAF) and analyzed with the MAFtools package in R software (Version: 4.3.1). Additionally, mafSurvival, a tool for analyzing survival analysis based on mutation status for each gene, was utilized. This further validated candidate variants in a separate cohort of 15 CRC patients undergoing Whole Exome Sequencing (WES). This additional validation step enhances the reliability of the initial findings and provides further evidence supporting the significance of *GPSM2* variants in CRC.

DNA extraction from the whole blood of 15 randomly selected patients from our cohort was performed using standardized procedures at ParsTous, Iran. The human whole exome was enriched using the “Agilent SureSelect V6 Target Enrichment Kit” (www.agilent.com) (accessed on 23 January 2024), following the manufacturer’s instructions. This process involved capturing genomic DNA through biotinylated RNA probes, designed to target exonic regions and include 10 base pairs (bp) of the flanking sequence. Subsequent amplification and sequencing were conducted on the Illumina HiSeq4000 platform (Illumina, Inc., Berlin, Germany). The data underwent analysis using conventional bioinformatics tools.

Variant calling was performed using the Genome Analysis Toolkit (GATK) software package 4.5.0.0 (https://gatk.broadinstitute.org) (accessed on 23 January 2024), which is proficient in identifying variations such as single-nucleotide mutations and small insertions/deletions (Indels) within a 20 bp range. The DNA sequence was then mapped and compared against the published human genome build (UCSC hg38 reference sequence). Variants with a minor allele frequency (MAF) of ≥0.1% for heterozygous variants or ≥1% for homozygous variants were excluded, using data from 1000 Genomes (Asian), Iranom, and the Genome Aggregation Database (gnomAD).

For the prediction of missense variants, Sorting Intolerant from Tolerant (SIFT) [26], Polymorphism Phenotyping, version 2 (PolyPhen2) [27] in conjunction with HumVar [27], and combined annotation-dependent depletion (CADD) with a Phred score of ≥20 were utilized [28]. These tools provided pre-computed predictions of the functional impact of human non-synonymous (change of amino acids) variants.

### 2.7. Survival Analysis

Survival analysis was performed using the mafSurvival package in R, which evaluates survival based on the specific mutation status of each gene. Also, Kaplan–Meier survival curves were generated for *GPSM2* using the survival, survminer, and ggplot2 R packages, as well as GraphPad Prism 10 [25]. The dataset was filtered based on a threshold of HR higher than 1 and Pvalue less than 0.05.

### 2.8. Receiver Operating Characteristic (ROC) Curve Analysis

Next, a generalized linear model and combined Receiver Operating Characteristic (ROC) curve analysis were applied to evaluate the diagnostic efficacy and construct diagnostic models. Parameters such as sensitivity, specificity, cut-off value, positive predictive value, negative predictive value, and area under the ROC curve were assessed to evaluate the discrimination power of individual or combined biomarkers. All procedures were carried out using the combioROC package in R.

### 2.9. Real-Time PCR Analysis

Next, total RNA was extracted from thirty fresh tissue samples following paraffinization using a Parstous kit. The quality and quantity of RNA were assessed using a Nanodrop 2000 spectrophotometer. cDNA was synthesized using a cDNA synthesis kit. Quantitative real-time PCR was conducted using specific primers for the *GPSM2* gene. The PCR was performed with the SYBR green master mix on an ABI-PRISM StepOne instrument. Gene expression data were normalized to GAPDH using a standard curve of cDNAs purchased from Quantitative PCR Human Reference RNA. The primer pair sequences used in this study are detailed in Table 1.

### 2.10. Correlation Analysis

We initially utilized the EMTOME database (http://www.emtome.org/) (accessed on 23 January 2024), a comprehensive resource for analyzing genes and signatures related to the epithelial-mesenchymal transition (EMT). This database is instrumental for diagnosing or preventing cancer metastasis. It enabled us to compile a list of genes associated with EMT. Subsequently, we examined the interactions between these genes and *GPSM2* using the STRING database.

Furthermore, we employed the GEPIA database, specifically its correlation analysis section, to explore the relationship between EMT and *GPSM2* genes within the TCGA dataset. Our analysis was focused on the COAD dataset.

In the subsequent step, we assessed the expression of the *GPSM2* gene and EMT-related genes using the qPCR method in the SW480 cell line. The outcomes of this correlation analysis were then analyzed using GraphPad Prism 10 software.

### 2.11. Statistical Analysis

The statistical analysis was conducted using SPSS 23.0, incorporating the application of the receiver operating characteristic (ROC), Kaplan–Meier survival analyses, independent sample t-tests, and χ^2^ tests. Univariate and multivariate analyses were performed based on Cox proportional hazard regression models. A *p*-value of less than 0.05 was deemed statistically significant.

## 3. Results

### 3.1. Patient Demographics

As outlined in the Materials and Methods section, this study focuses on two distinct patient groups. The first group includes TCGA-COAD patients, comprising data from 250 COAD tissue samples. This dataset includes 17 non-cancerous samples and 233 cancerous samples from the white population within the TCGA database. After excluding patients with missing clinical data, the demographic information of 214 patients was analyzed using SPSS (Version 28) and is presented in Table 2A. Of these, 48.1% were female, and the remaining 51.9% were male. Regarding disease stages, approximately 54.9% of the participants were diagnosed with stages 1 and 2, 31.4% with stage 3, and 13.7% with stage 4.

For the second cohort, initially, 120 CRC cases were selected following histological examination by two pathologists. Exclusion criteria included hereditary colorectal cases and those who chose not to participate, resulting in a final cohort of 64 individuals (refer to Table 2B).

### 3.2. FireBrowse Database Demonstrated the Differential Expression Pattern of GPSM Family across Different Cancers

**Figure 1:**
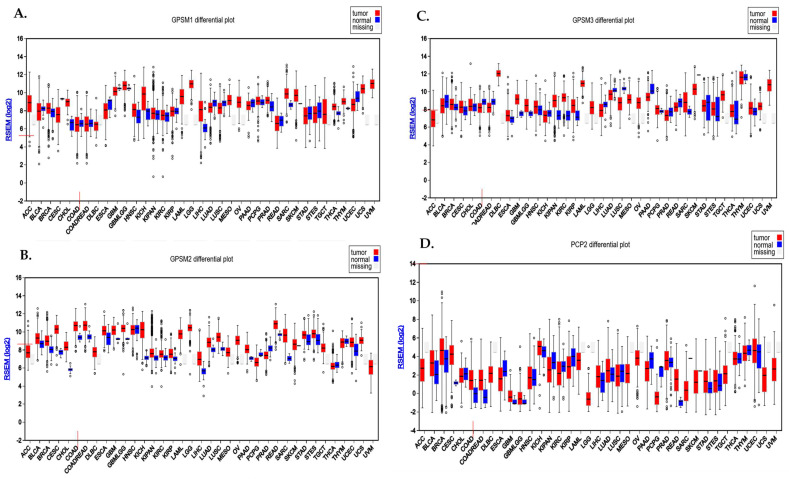
Differential expression pattern of GPSMs in CRC using FireBrowse database. (**A**) GPSM1, (**B**) GPSM2, (**C**) GPSM3, and (**D**) GPSM4 differential expression among all cancers.

Analysis conducted using the FireBrowse database demonstrated the differential expression pattern of GPSMs among different cancers including CRC. Notably, GPSM1 and GPSM3 exhibited a downregulation, whereas *GPSM2* and *PCP2* displayed overexpression in CRC as depicted in Figure 1A.

### 3.3. UALCAN Demonstrated the Expression Levels of GPSMs in CRC

**Figure 2:**
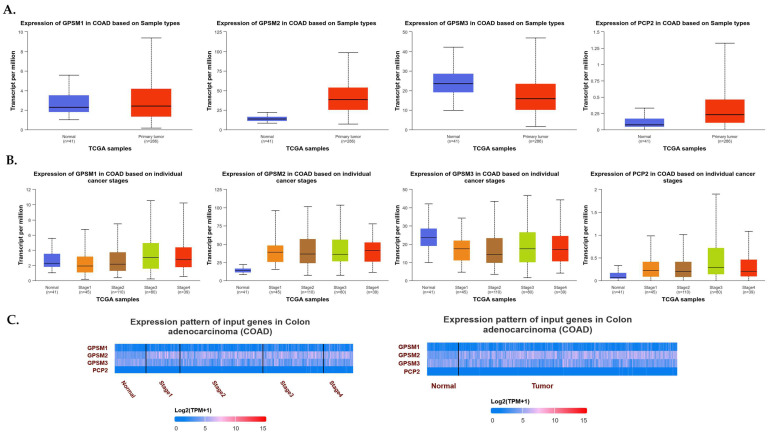
(**A**) Expression levels of *GPSM*1, *GPSM2*, *GPSM3*, and *PCP2* across healthy controls and CRC patients. (**B**) Expression levels of *GPSM1*, *GPSM2*, *GPSM3*, and *PCP2* across different stages of CRC. (**C**) Heatmap representing the differential expression patterns of the GPSM family members across different stages of CRC and compared to healthy controls.

To explore the expression dynamics of the GPSM gene family in CRC, we utilized the UALCAN database. Our comprehensive analysis aimed to elucidate the expression levels of *GPSM1*, *GPSM2*, *GPSM3*, and *PCP2* across healthy controls and CRC patients, as well as to discern the gene expression patterns across different stages of CRC. As depicted in Figure 2A, our findings revealed a downregulation of *GPSM1* and *GPSM3* and an overexpression of *GPSM2* and *PCP2*, corroborating data from the FireBrowse database. A detailed examination of differential expression across various stages of CRC indicated that *GPSM2* and *PCP2* continued to show overexpression compared to healthy controls. Interestingly, *GPSM3* also exhibited downregulation across different stages versus healthy controls. However, *GPSM1* appeared to be up-expressed in advanced stages, which may suggest a compensatory mechanism or stage-specific role.

To visually represent the differential expression patterns of the GPSM family members, we constructed a heatmap (Figure 2B), which provided a clear and succinct visual representation of the changes in gene expression across different stages of CRC and compared to healthy controls.

### 3.4. Comprehensive Examination of RNA Sequencing Data Revealed GPSM2 as an Interesting Candidate

To conduct a comprehensive analysis of the differential expression profiles of the GPSM family members within our TCGA dataset, we performed gene expression analysis on a total of 214 CRC cases and 17 healthy controls from the white population. Initially, we categorized the patients into three distinct subgroups based on tumor stage, grouping stages 1 and 2 together and stages 3 and 4 into another subgroup. We then utilized the DESeq2 package to carry out differential expression analysis within these subgroups separately, adhering to stringent criteria: an adjusted *p*-value of less than 0.05 and a log2 fold change (|logFC|) of greater than or equal to 1.5.

Upon completion of this analysis, our subsequent screening identified *GPSM*2 as the sole member of the GPSM family exhibiting differential expression patterns across all three subgroups of our dataset. This discovery highlights the potential of GPSM2 as a biomarker for CRC diagnosis and prognosis, given its consistent altered expression across different stages of the disease.

### 3.5. Pan-Cancer Analysis among GI Cancers Suggested GPSM2 as a Potential Prognostic Biomarker

**Figure 3:**
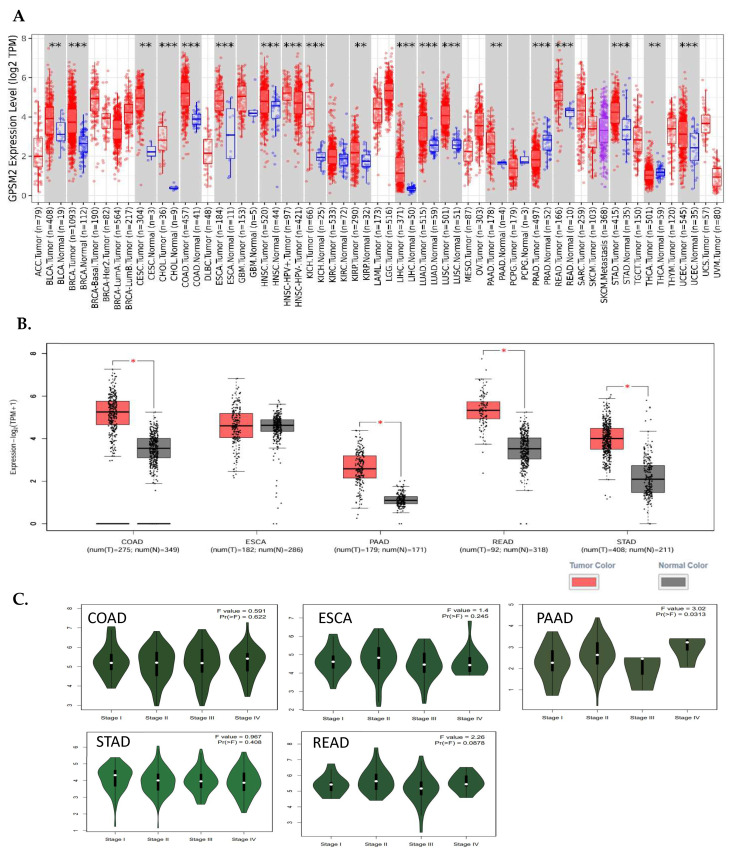
Differential expression of *GPSM*2 in different cancers. (**A**) TIMER2 DE analysis results indicative of significant upregulation of *GPSM*2 in all GI cancers (**: *p*-value < 0.01; ***: *p*-value < 0.001). (**B**) GEIPA2 DE results in GI cancers using TCGA and GTEx datasets. * in bars shows the significance of upregulation. (**C**) The violin plots by the “stageplot” module in GEPIA2 in different GI cancers show the *GPSM*2 expression in different stages of each cancer.

The analysis of TIMER2 revealed that *GPSM*2 is significantly overexpressed in all gastrointestinal (GI) cancers, including colorectal adenocarcinoma (COAD), esophageal squamous cell carcinoma (ESCA), pancreatic adenocarcinoma (PAAD), renal cell carcinoma (READ), and stomach adenocarcinoma (STAD) (Figure 1). Additionally, the GE-PIA2 analysis indicated that *GPSM2* is significantly upregulated in COAD, PAAD, READ, and STAD. In the pathological stage analysis conducted with GEPIA2, a stage-specific change was observed in PAAD with a *p*-value less than 0.05 (Figure 3).

**Figure 4:**
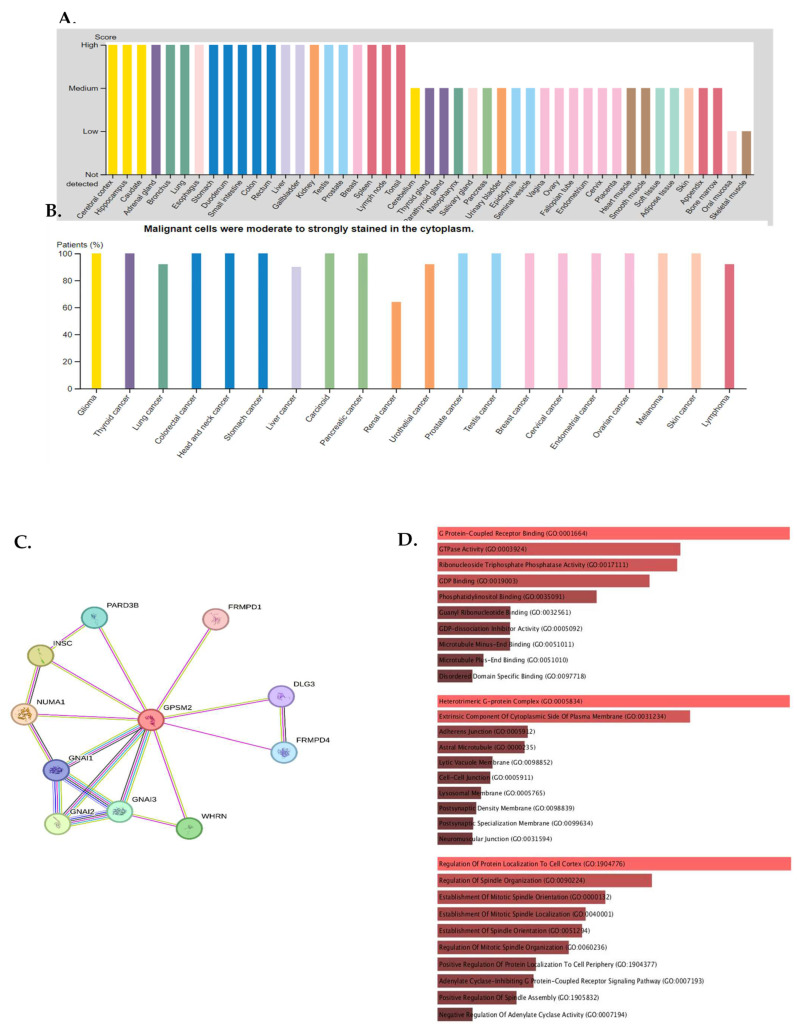

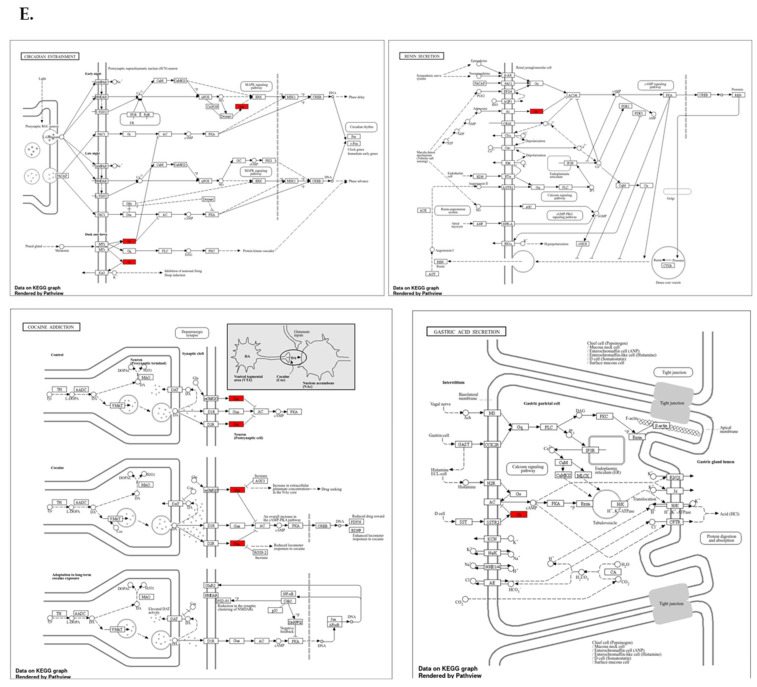
Protein expression analysis and enrichment analysis results. (**A**) *GPSM*2 expression in different normal tissues is indicated which is scored low to high. (**B**) The percentage of patients with high and moderate *GPSM*2 protein expression in each cancer is shown in the bar plot. (**C**) Constructed PPI network of *GPSM2* by STRING. (**D**) Functional enrichment analysis by Gene Ontology (GO), each bar graph is indicative of one GO term enrichment. (**E**) KEGG pathway enrichment results. Enriched pathways with the highest value are indicated.

The expression of *GPSM2* protein in normal gastrointestinal tissues, as well as in GI cancers, was examined using the Human Protein Atlas (HPA) database. The results showed a high level of *GPSM2* in all GI tissues, as depicted in Figure 4A. Figure 4B illustrates the percentage of patients with high and moderate *GPSM2* protein expression.

To investigate the interaction of the *GPSM2* protein, a STRING analysis was performed, and the constructed protein–protein network is shown in Figure 4C. The identified genes were enriched using Enrichr to elucidate the function of the protein network. The functional enrichment results highlighted significant functions of the network. In GO ontology enrichment, the most important functions enriched were the regulation of protein localization in biological processes (BPs), protein-coupled receptor binding in molecular functions (MFs), and the heterodimeric G-protein complex in cellular components (CCs) (Figure 4D). Furthermore, in KEGG pathway enrichment, gastric acid secretion, cocaine addiction, renin secretion, and regulation of lipolysis in adipocytes were enriched with the highest *p*-values (Figure 4E). These discoveries emphasize the critical role of the *GPSM2* protein and its protein network in GI cancers.

**Figure 5:**
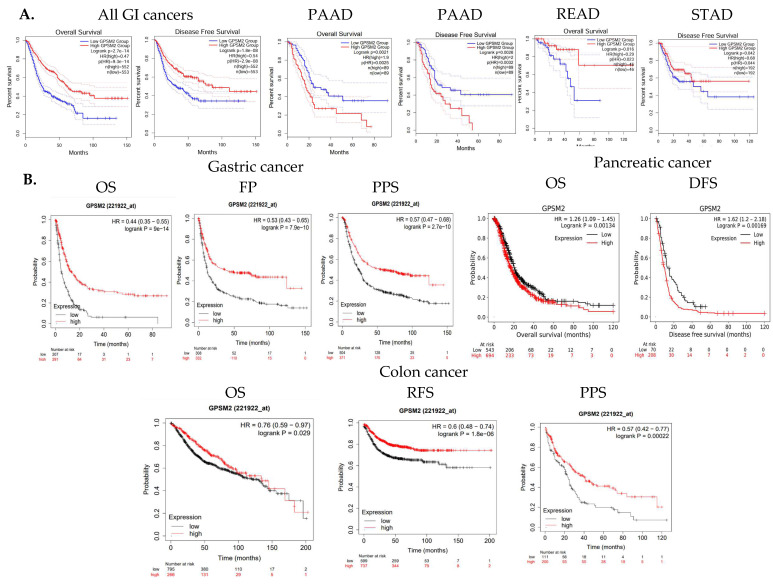
Survival analysis results. (**A**) OS and DFS of GI cancer patients related to *GPSM*2 expression included in the TCGA database performed using the GEPIA2 database. Significant (*p* value < 0.05) Kaplan–Meier plots are indicated that show the correlation between *GPSM2* expression and OS or DFS of patients in each cancer. (**B**) Associations between *GPSM2* expression and survival prognosis of patients were further assessed using the Kaplan–Meier plotter tool in different GEO datasets. Different survival analyses including OS, PF, PPS, and DFS were performed for colon cancer, gastric cancer, and pancreatic cancer.

The overall survival (OS) and disease-free survival (DFS) of GI cancer patients, including COAD, ESCA, READ, and STAD, were analyzed in the TCGA database using GEPIA2. The results indicated that high levels of *GPSM2* expression are associated with shorter overall survival and disease-free survival in all GI patients (Figure 3A). Moreover, overexpression of *GPSM2* was linked to poor OS in patients with PAAD (*p* = 0.002) and READ (*p* = 0.02) in TCGA cohorts. Additionally, a high expression of *GPSM2* was correlated with poor DFS prognosis in PAAD (*p* = 0.003) and STAD (*p* = 0.04) (Figure 5A).

Further analysis using the Kaplan–Meier plotter tool demonstrated that a high expression of *GPSM2* is associated with low OS (*p* = 9 × 10^−14^), false-positive (FP) results (*p* = 7.9 × 10^−10^), and progression-free survival (PPS) (*p* = 2.7 × 10^−10^) in gastric cancer patients (Figure 5B). A high expression of *GPSM2* was also correlated with shorter OS (*p* = 0.001) and DFS (*p* = 0.001) in patients with pancreatic cancer (Figure 3C). Moreover, *GPSM2* overexpression was linked to OS (*p* = 0.02), PPS (*p* = 0.0002), and relapse-free survival (RFS) (*p* = 1.8 × 10^−6^) in colon cancer patients (Figure 5B).

**Figure 6:**
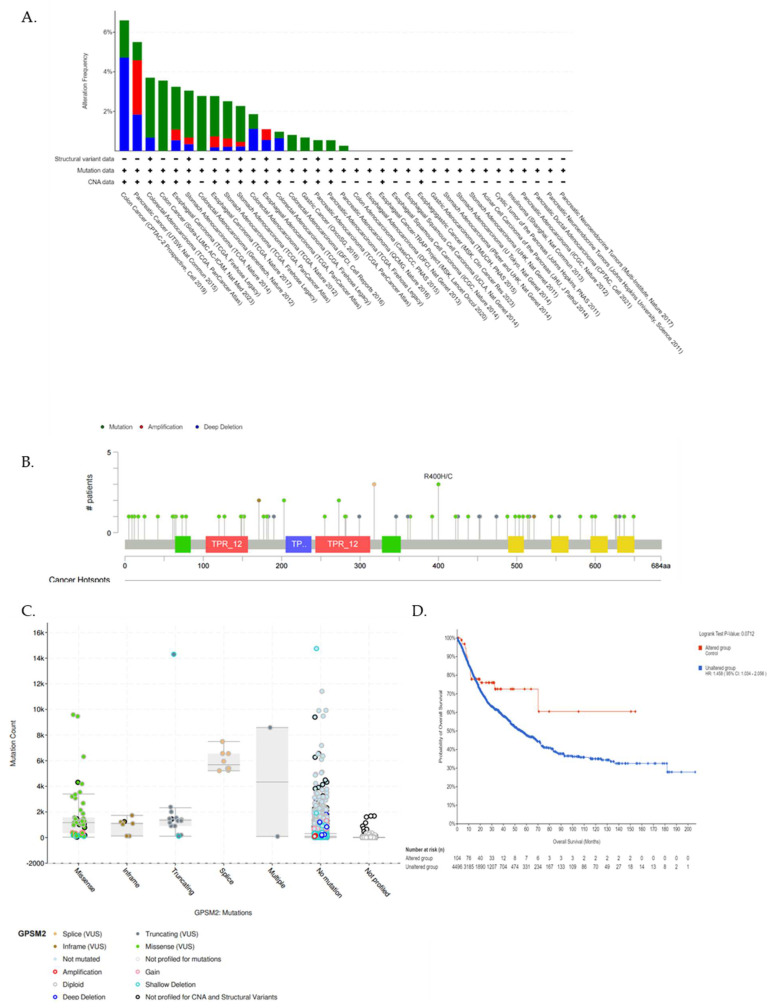
Analysis of genetic alterations in *GPSM*2 and its association with GI cancers using cBioPortal database. (**A**) Genetic alteration frequency in different GI datasets. The colors of the bars are indicative of the type of alterations. (**B**) Lollipop plot of *GPSM2* protein and its mutation sites; also, the mutation frequency is indicated. The Y axis shows the amino acid number and the position of frequent mutations in the *GPSM2* protein structure. (**C**) Bar plot of mutation type frequency which indicates the types of mutations in *GPSM2* in GI cancers. (**D**) The correlation between OS and *GPSM2* genetic alterations.

We utilized the cBioPortal database further to underscore the *GPSM2* genetic alterations in GI cancers. Figure 6A represents the frequency of different genetic alterations including mutations, amplifications, and deep deletions in defined cancers. Results showed that the most frequent type of genetic alterations of *GPSM2* in GI cancers was “Mutations” in almost all of the datasets, then “Deep deletions” was the second type of frequent alterations in GI cancers. “Amplification” alterations were observed in stomach adenocarcinoma, esophageal carcinoma, and pancreatic adenocarcinoma (Figure 6A). Also, results showed that missense mutations in *GPSM2* were the most prevalent type of mutation in GI cancers (Figure 6B,C). However, we did not observe any significant (*p* < 0.05) correlation between *GPSM*2 genetic alterations and the survival of GI patients (Figure 6D).

**Figure 7:**
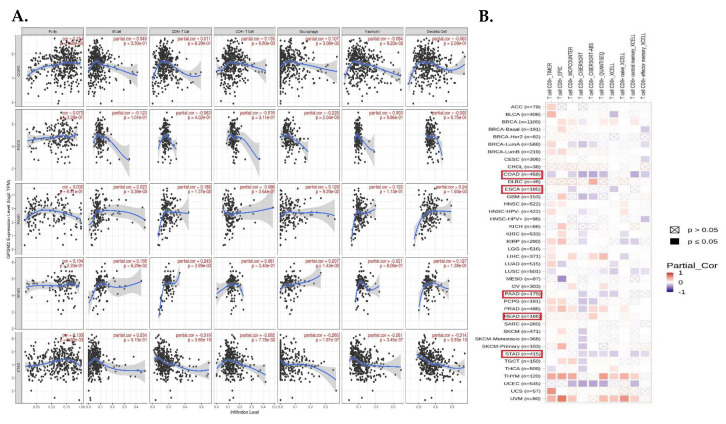
Analysis of immune infiltration association with *GPSM2* expression in GI cancers. (**A**) scatter plots obtained with TIMER that show the significance of the correlation of *GPSM2* expression level in GI cancers. (**B**) TIMER2 database was used to apply different algorithms to explore the potential correlation between the expression level of *GPSM2* and the level of CD8+ T cell infiltration in GI cancers in TCGA.

Tumor-infiltrating cells including CD4+ T cells, CD8+ T cells, macrophages, neutrophils, B cells, and dendritic cells have crucial effects on tumor initiation and progression, so we evaluated the correlation between the expression level of *GPSM2* and the level of tumor-infiltrating cells in GI cancers. The scatter plots obtained with the TIMER database demonstrated that the expression level of *GPSM2* was significantly related to high levels of immune cell infiltration in GI cancers (Figure 7A). For instance, the correlation between the *GPSM2* expression and level of infiltrating CD4+ T cells and macrophages in COAD and with the macrophage level in ESCA was observed. Also, this significant correlation was observed in PAAD for B cells, CD8+ T cells, and dendritic cells; in READ for CD8+ T cells; and in STAD for CD8+ T cells, macrophages, neutrophils, and dendritic cells (Figure 5A). Moreover, in the heatmap plot obtained by the TIMER2, we observed that the expression of *GPSM2* is negatively correlated with the CD8+ T cell infiltration in COAD and STAD based on most algorithms and also in PAAD and ESCA based on some algorithms (Figure 7B).

### 3.6. DNA-Seq and Whole Exome Sequencing

Our previous research has underscored the prevalence of missense mutations, especially single-nucleotide polymorphisms (SNPs), throughout both the early and late stages of CRC. A substantial number of patients were identified with mutations in their APC or TP53 genes, which are frequently linked to Wnt/B-catenin signaling, genome integrity, and MAPK signaling pathways. Moreover, we established connections between mutated genes and drugs targeting tyrosine kinase, transcription factor complex, DNA repair, and other related processes. The involvement of mutated genes in various carcinogenic signaling pathways, including RTK-RAS, Wnt, Hippo, Notch, and others, was further clarified [29]. Moreover, DNAsequencing of TCGA MAF data revealed several pathogenic variants in our dataset which are demonstrated in Table 3.

To validate the gene variants related to *GPSM2* in our patient samples, whole exome sequencing was conducted on 15 cancer samples, revealing no pathogenic or likely pathogenic variants in this gene. This finding aligns with analyses performed on TCGA-COAD MAF data, suggesting that the high mutation rate in *GPSM2*, possibly due to its non-pathogenic nature, does not impact survival outcomes.

### 3.7. Survival Analysis

**Figure 8:**
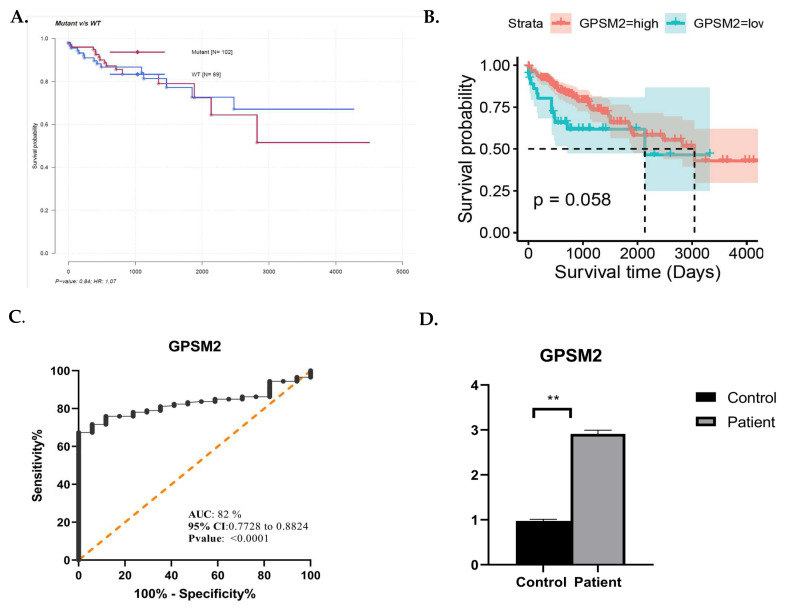
(**A**) Mafsurvival analysis of *GPSM2*. (**B**) Kaplan–Meier visualization of *GPSM2* using Survival, survminer, and ggplot2 R packages from R software v4.2.2. (**C**) ROC curve analysis using GraphPad Prism. (**D**) Real-time analysis showing the overexpression of *GPSM2* in patients vs. control. * represents each death event. ** represents *p*-value <0.05.

In this study, we also performed survival analysis using the mafSurvival package in R, which evaluates survival based on the specific mutation status of each gene. This analysis highlighted the *GPSM2* gene as having one of the highest classified mutation rates in CRC. Interestingly, despite the high mutation rate in this gene, as shown in Figure 8A, individuals with mutations in this gene exhibited better survival rates compared to those without mutations. Further analysis and survival assessment were conducted using the survival, survminer, and ggplot2 R packages, as depicted in Figure 8B.

### 3.8. ROC Curve Analysis Highlights the Potential Diagnostic Ability of GPSM2

ROC curve analysis was performed using GraphPad prism 9.0.0. as depicted in Figure 8C. The area under the curve (AUC) for *GPSM2* was 0.82, accompanied by a confidence interval (95% CI) and sensitivity and specificity rates of 0.77 and 0.88, respectively, which aligns with the criteria for an effective diagnostic marker.

### 3.9. The Expression Level of GPSM2 in Additional Cohort

To substantiate the significance of *GPSM2* as a diagnostic and prognostic marker in CRC, we conducted an independent validation using an additional cohort including 64 CRC cases and matched normal controls. This validation involved quantitative real-time PCR (qRT-PCR) to assess the mRNA expression levels of *GPSM2* within the tumor samples. The resulting data indicated a marked increase in the expression of *GPSM2* in CRC tissues versus controls, with a statistically significant difference observed (*p* < 0.05) as shown in Figure 8D.

To further analyze the correlation between the *GPSM2* gene and EMT-related genes, the expression levels of these genes were also assessed in the SW480 cell line using quantitative PCR (qPCR).

### 3.10. Correlation Analysis Revealed GPSM2 with a Potential Role in EMT Process

**Figure 9:**
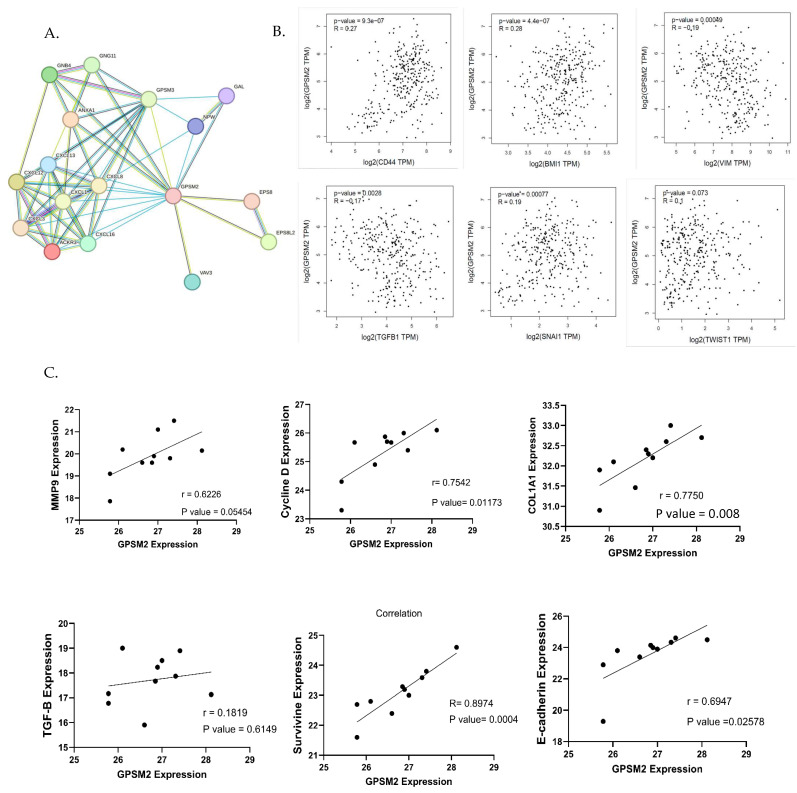
Correlation analysis. (**A**) String network demonstrating the interaction between EMT genes downloaded from EMTome and *GPSM2*. (**B**) The correlation analysis between EMT genes and *GPSM2* using the GEPIA2 database. (**C**) Correlation analysis between several EMT genes and *GPSM2* expression in SW480 cell line using GraphPad Prism 10.

To identify genes associated with the EMT process, the EMTOME database was utilized. Subsequently, the STRING database was employed to analyze the interactions between these genes and *GPSM2*, as illustrated in Figure 9A. Additionally, a correlation analysis between EMT genes including *CD44*, *BMI1*, *VIM*, *TGFb1*, *SNAI1*, and *TWIST1* and *GPSM2* genes was conducted using the TCGA-COAD dataset through the GEPIA2 database. This analysis revealed a positive correlation between *GPSM2* and *CD44*, *BMI1*, *SNAI1*, and *TWIST1* genes, as well as a negative correlation with VIM and TGFb1 genes (Figure 9B). To validate these findings in vitro, an expression correlation analysis was performed, comparing the expression of EMT genes, including *MMP9*, *E-cadherin*, *Cyclin D*, *Col1A1*, *Survivin*, and *TGFb1*, with *GPSM2*. The results were visualized using GraphPad Prism 10 software. The analysis demonstrated a positive correlation between all mentioned genes with *GPSM2*, although *TGF-b1* demonstrated an insignificant and low Pearson correlation coefficient (r = 0.18) (Figure 9C).

This finding suggests that *GPSM2* has a positive correlation with multiple EMT genes, indicating a potential involvement in the EMT process. However, its correlation with *TGF-b1* is weak and lacks statistical significance.

## 4. Discussion

GPSMs represent a class of proteins that play a pivotal role in the development of various types of tumors, including breast cancer [13,14]. They play a crucial role in the regulation of G protein-coupled receptors, which are essential for the development of these tumors [14]. The GPSM family is part of the class of type two AGS proteins found in mammals, which play a vital role in the Gi/Go/transducin family of G proteins. These proteins function as guanine nucleotide dissociation inhibitors [29].

*GPSM1*, the first member of the GPSM family, is renowned for its crucial role in regulating GPCR signaling and various cellular functions. These functions encompass asymmetric cell division, autophagy, intracellular pathogen clearance, protein trafficking, behavioral changes associated with addiction, polycystic kidney disease, and chemotaxis [30,31,32,33,34,35]. Zhang et al.’s research underscores the pivotal role of *GPSM1* downregulation in BALL-1 and Reh cells in the development of B-ALL. This downregulation plays a crucial role in the pathogenesis of B-ALL by reducing cell proliferation, hindering cell cycle progression, and promoting apoptosis through the modulation of the ADCY6-RAPGEF3-JNK pathway. As a result, *GPSM1* emerges as a promising target for B-ALL treatment [36].

*GPSM3*, also known as *AGS4* or *G18*, is a regulator of GPCR (G-protein coupled receptor) and G protein function. It plays a crucial role in influencing the invasive or migratory phenotypes of various cancers. Additionally, it affects how these cancers respond to survival or angiogenic factors, which may be secreted as tumors progress [37]. Recent studies indicate that *GPSM3* may play a crucial role in treating inflammatory diseases and cancer, particularly in the context of glioblastoma multiforme (GBM). Specifically, *GPSM3* has emerged as a promising target for immunotherapy in GBM patients, due to its strong correlation with immune checkpoints and tumor microenvironment (TME) immunosuppressors in GBM [38].

While research on the impact of *PCP2* in cancer is limited, it has been proposed as a potential therapeutic target for neuropathic pain associated with head and neck cancer. Additionally, bioinformatics studies have highlighted a connection between this gene and reduced survival rates in breast and prostate cancer [14].

Research has demonstrated that *LGN*/*GPSM2* is significantly upregulated in breast cancer cells, playing a crucial role in cytokinesis. Furthermore, it appears that *GPSM2*, through the “PBK/TOPK-LGN/GPSM2” pathway, can significantly contribute to cell growth, making it a potential target for cancer therapy [39]. Several studies have highlighted the significance of *GPSM2* in cancer development. A study by Zhou et al. specifically examined the role of the *GPSM2* gene in pancreatic adenocarcinoma (PAAD) [19]. This research demonstrated that *GPSM2* overexpression in PAAD is associated with a history of chronic pancreatitis, tumor staging, and tumor grade. Additionally, the gene’s involvement in cell migration and immune cell infiltration within the tumor microenvironment was emphasized, considering *GPSM2* as a prognostic marker and potential therapeutic target in PAAD [19]. Another study involving pancreatic CD133+ stem cells from the PANC-1 cell line suggested that *GPSM2* plays a regulatory role and influences the proliferation and migration of these cells [18].

The expression of the *GPSM2* gene has also been investigated in liver cancer, which demonstrated a correlation between *GPSM2* overexpression and liver cancers associated with hepatitis B virus (HBV) as well as hepatocellular carcinoma cell lines. The elevated expression of *GPSM2* is linked to larger tumor sizes and HBV infection. Given the gene’s pivotal role in cell growth, cell cycle regulation, migration, and invasion via the phosphatidyl 3-kinase/protein kinase signaling pathway, *GPSM2* is proposed as an oncogene and therapeutic target in liver cancer [17,40].

Correlations between *GPSM2* and the clinical features of breast cancer patients have been documented. Additionally, the upregulation of both *DYNC1I1* and *GPSM2* genes has been associated with reduced patient survival. Other research has highlighted GPSM2’s association with drug resistance in breast cancer and its potential as a therapeutic target to enhance drug sensitivity, particularly concerning chemotherapy drugs such as paclitaxel [21]. In contrast, a decline in *GPSM2* gene expression in non-small cell lung cancer (NSCLC) suggests a protective effect of this gene against cancer metastasis. Induction of EMT by activating the ERK/GSK-3β/Snail pathway, which silences the *GPSM2* gene, has been implicated in NSCLC metastasis. Further studies on lung adenocarcinoma have demonstrated that the downregulation of *GPSM2* accelerates cell proliferation through the EGFR pathway [41].

Recent findings emphasizing the role of GPSMs in cancer have led to a focus on investigating this family in CRC. Utilizing the FireBrowse online database, the expression patterns of (*GPSM1-4*) were examined in CRC samples, revealing the decreased expression of *GPSM1* and *GPSM3* and increased expression of *GPSM2* and *PCP2*. Comparative expression analysis in CRC versus healthy individuals across different cancer stages was in line with initial observations. Notably, *GPSM1* expression increased in advanced-stage CRC compared to early-stage cases. Heatmap analysis using the UALCAN database generally indicated low expression levels of *GPSM1* and *PCP2* in CRC, with *GPSM2* showing the highest expression. To further examine these genes, data from TCGA were analyzed using RNA sequencing, comparing gene expression across early-stage (stages 1 and 2), stage 3, and stage 4 CRC. The *GPSM2* gene exhibited significant differential expression across all three groups, suggesting its potential as a biomarker for the prognosis of patients with CRC. However, it is important to mention that we focused exclusively on the white population within the TCGA dataset, ensuring that our results represented this subgroup.

Subsequent studies on the *GPSM2* gene in gastrointestinal cancers included a pan-cancer analysis across ESCA, PAAD, READ, and STAD cancers, demonstrating significant *GPSM2* overexpression across all. Protein expression assays confirmed high levels of *GPSM2* in all gastrointestinal tissues. GO ontology enrichment analysis highlighted the regulation of protein localization in biological processes (BPs), protein-coupled receptor binding in molecular functions (MFs), and G protein heterodimeric complex in cellular components (CCs) as key functions enriched in the *GPSM2* protein network. KEGG pathway enrichment analysis revealed significant roles in fat cells, including gastric acid secretion, cocaine addiction, renin secretion, and lipolysis regulation. These findings underscore the importance of the *GPSM2* protein and its associated protein network in gastrointestinal cancers.

ROC curve analysis suggested that *GPSM2* could serve as a potential diagnostic marker in CRC with a diagnostic power of 82%. Prognostic analysis, including GEPIA2, Kaplan–Meier, and MAF survival analyses, generally indicated that higher *GPSM2* expression levels correlate with shorter overall and disease-free survival in gastrointestinal patients. Overexpression of *GPSM2* was associated with poor prognosis in PAAD and READ within the TCGA cohorts. High *GPSM2* expression was also linked to unfavorable prognosis in terms of disease-free survival in PAAD and STAD. Additionally, *GPSM2* overexpression correlated with lower overall survival, progression-free survival, and pathologic complete response of gastric cancer patients. These observations underscore the diagnostic and prognostic relevance of *GPSM2* in gastrointestinal cancers, including CRC.

Genomic alterations in the *GPSM2* gene were also examined in gastrointestinal cancers using the cBioPortal database, revealing that mutations and deep deletions were the most common types of genetic alterations. Missense mutations predominantly occur in gastrointestinal cancers. However, WES analysis revealed no significant and pathogenic or likely pathogenic genetic variations in *GPSM2* in our patients. The expression of *GPSM2* was also found to be significantly associated with high levels of immune cell infiltration in gastrointestinal cancers; however, no significant correlation was detected between *GPSM2* expression and survival rates or immune cell infiltration in CRC. Analysis of *GPSM2* expression concerning tumor purity demonstrated highly positive correlation and correlations with the infiltration levels of CD4+ T cells and macrophages within CRC.

The positive correlation between *GPSM2* and most EMT genes suggests a potential role for this gene in the EMT process. However, further research is necessary to substantiate this hypothesis. Ultimately, despite the study’s limitations, including the absence of in vivo and functional studies, this research makes a significant contribution by being the first to explore the importance of *GPSM2* at both the RNA and DNA levels.

## 5. Conclusions

In conclusion, our study suggests that *GPSM2* could serve as a novel prognostic marker in CRC. Despite previous efforts to understand the function of this gene in CRC, our research lacks a functional study to validate *GPSM2’s* role in this context. Therefore, further investigation is necessary to fully understand the significance of *GPSM2* in CRC and to develop potential therapeutic strategies targeting this gene.

## Figures and Tables

**Table 1:** Primer pairs used in the study.

Gene Name	Primer Sequence
*GPSM2-F*	GGGAAGCGAAAGCTAGTG
*GPSM2-R*	CTTGCTTCTCCCACCTTG
*GAPDH F*	ATCAGCAATGCCTCCTGCAC
*GAPDH R*	TGGTCATGAGTCCTTCCACG

**Table 2:** Demographic information of individuals who participated in (A) the COAD TCGA dataset and (B) the patients who participated in the study.

**A.**		**B.**	
**Characteristic**	**Patients (%)**	**Characteristic**	**Patients (%)**
**Age**	65.76 ± 13.01	**Age**	55.34 ± 13.67
**Sex**		**Sex**	
Female	103 (48.1)	Female	35 (54.7)
Male	111 (51.9)	Male	29 (45.3)
**TMN classification**		**TMN classification**	
Stage I–II	112 (54.9)	Stage I–II	37 (57.8)
Stage III	64 (31.4)	Stage III	25 (39.1)
Stage IV	28 (13.7)	Stage IV	2 (3.1)
**Tumor size**		**Grade**	
T1	8 (3.7)	Poorly-differentiated	29 (45.3)
T2	33 (15.4)	Moderately-differentiated	34 (53.1)
T3	145 (67.8)	Well-differentiated	1 (1.6)
T4	28 (13.1)	Undifferentiated	0 (0)
**Nodal status**		**Nodal status**	
Yes	93 (43.5)	Yes	28 (43.7)
N1	59 (27.7)		
N2	34 (15.8)		
No	121 (56.5)	No	36 (56.3)
**Distant metastasis**		**Distant metastasis**	
Yes	28 (15.8)	Yes	1 (1.6)
No	150 (84.2)	No	63 (98.4)

**Table 3:** Novel pathogenic variants in COL9A1 identified by analyzing TCGA SNV file.

Hugo_Symbol	Chromosome	Start_Position	End_Position	Strand	Variant_Classification	Variant_Type	Tumor_Seq_Allele1	Tumor_Seq_Allele2	dbSNP_RS
*GPSM2*	chr1	108,898,897	108,898,897	+	Nonsense_Mutation	SNP	G	T	NA
*GPSM2*	chr1	108,901,836	108,901,836	+	Missense_Mutation	SNP	G	A	rs753366137
*GPSM2*	chr1	108,918,769	108,918,769	+	Nonsense_Mutation	SNP	C	T	rs776770855
*GPSM2*	chr1	108,929,776	108,929,776	+	Frame_Shift_Del	DEL	T	-	novel
*GPSM2*	chr1	108,901,888	108,901,888	+	Frame_Shift_Del	DEL	A	-	NA
*GPSM2*	chr1	108,918,769	108,918,769	+	Nonsense_Mutation	SNP	C	T	rs776770855
*GPSM2*	chr1	108,903,125	108,903,125	+	Splice_Site	SNP	G	T	NA

## Data Availability

The data that support the findings of this study are available on request from the corresponding author upon reasonable request.
